# Increased Urinary Angiotensin-Converting Enzyme 2 in Renal Transplant Patients with Diabetes

**DOI:** 10.1371/journal.pone.0037649

**Published:** 2012-05-22

**Authors:** Fengxia Xiao, Swapnil Hiremath, Greg Knoll, Joseph Zimpelmann, Kajenny Srivaratharajah, Deepak Jadhav, Dean Fergusson, Chris R. J. Kennedy, Kevin D. Burns

**Affiliations:** Division of Nephrology, Department of Medicine, Ottawa Hospital Research Institute, Kidney Research Centre, University of Ottawa, Ottawa, Ontario, Canada; Max-Delbrück Center for Molecular Medicine (MDC), Germany

## Abstract

Angiotensin-converting enzyme 2 (ACE2) is expressed in the kidney and may be a renoprotective enzyme, since it converts angiotensin (Ang) II to Ang-(1-7). ACE2 has been detected in urine from patients with chronic kidney disease. We measured urinary ACE2 activity and protein levels in renal transplant patients (age 54 yrs, 65% male, 38% diabetes, n = 100) and healthy controls (age 45 yrs, 26% male, n = 50), and determined factors associated with elevated urinary ACE2 in the patients. Urine from transplant subjects was also assayed for ACE mRNA and protein. No subjects were taking inhibitors of the renin-angiotensin system. Urinary ACE2 levels were significantly higher in transplant patients compared to controls (p = 0.003 for ACE2 activity, and p≤0.001 for ACE2 protein by ELISA or western analysis). Transplant patients with diabetes mellitus had significantly increased urinary ACE2 activity and protein levels compared to non-diabetics (p<0.001), while ACE2 mRNA levels did not differ. Urinary ACE activity and protein were significantly increased in diabetic transplant subjects, while ACE mRNA levels did not differ from non-diabetic subjects. After adjusting for confounding variables, diabetes was significantly associated with urinary ACE2 activity (p = 0.003) and protein levels (p<0.001), while female gender was associated with urinary mRNA levels for both ACE2 and ACE. These data indicate that urinary ACE2 is increased in renal transplant recipients with diabetes, possibly due to increased shedding from tubular cells. Urinary ACE2 could be a marker of renal renin-angiotensin system activation in these patients.

## Introduction

Angiotensin-converting enzyme 2 (ACE2) is a recently identified member of the renin-angiotensin system (RAS) that degrades angiotensin (Ang) II to the seven amino acid peptide fragment Ang-(1-7) [1], [2]. ACE2 is a homologue of angiotensin-converting enzyme (ACE), but is not blocked by ACE inhibitors. Although ACE2 is found in many tissues, expression is especially high in the kidney, particularly within cells of the proximal tubule [3]–[5]. In mice deletion of the ACE2 gene is associated with development of late-stage glomerulosclerosis, and acceleration of diabetic nephropathy, in the absence of hypertension [6], [7]. In spontaneously hypertensive rats, administration of human recombinant ACE2 reduces blood pressure [8], and in diabetic mice, exogenous human ACE2 diminishes blood pressure and glomerular injury [9]. Thus, ACE2 may be an endogenous protector against the progression of chronic kidney disease (CKD).

In kidney tubular epithelial cells, ACE2 is localized to the apical membrane and also appears in the cytoplasm [3], [10]. ACE2 is shed at its carboxy-terminus from the plasma membrane in cultured human embryonic kidney cells and airway epithelial cells, a process catalyzed by the enzyme “a disintegrin and metalloproteinase-17” (ADAM-17) [11]–[13]. Whether this process occurs in the proximal tubule is unclear, although soluble ACE2 has been detected in human urine [10]. In a recent study, urinary levels of ACE2 protein were significantly increased in humans with CKD (the majority with chronic glomerulonephritis), compared to healthy controls, as determined by enzyme-linked immunosorbent assay (ELISA) [14]. Urinary ACE2 was also higher in diabetics with CKD [14]. These results suggest that ACE2 may be shed into the urine, and could be a biomarker in CKD patients. However, the presence of urinary ACE2 has not been studied in renal transplant recipients, and the factors associated with elevated urinary ACE2 remain unclear. Accordingly, the principle objective of the present study was to determine if urinary ACE2 activity, protein, and mRNA can be detected in renal transplant patients, and to identify factors associated with the presence of ACE2. In addition, we examined factors associated with urinary ACE activity, protein and mRNA in these patients. Our data indicate that urinary ACE2 is increased in renal transplant recipients with diabetes, possibly due to increased shedding from tubular cells.

## Methods

### Ethics Statement

This study involved recruitment of human subjects as described below, with written informed consent, and the study was conducted according to the principles expressed in the Declaration of Helsinki. The study protocols were approved by the Research Ethics Board of The Ottawa Hospital (protocol numbers 200951201H, 200568201H).

### Study Subjects

The subjects in this study were 50 healthy controls (age >18 yrs), recruited from the hospital or research centre staff, with no history of kidney disease, hypertension, or diabetes, and 100 renal transplant recipients from The Ottawa Hospital Renal Transplant Program, age >18 yrs, and >3 months post-transplant. At the time of enrollment, half of the transplant subjects (n = 50) were also enrolled in an ongoing randomized controlled trial to determine the effect of the ACE inhibitor ramipril on transplant outcomes (ACE inhibition for the preservation of renal function and survival in kidney transplantation; International Standard Randomized Controlled Trial Number Registry ISRCTN-78129473), but had not yet received either placebo or ramipril. These subjects had documented significant proteinuria (>200 mg urinary protein/day) at baseline. The 50 other transplant recipients were patients without significant proteinuria (spot urine ACR in the normal range, or <200 mg proteinuria/day). Subjects were excluded if they were taking ACE inhibitors, angiotensin receptor antagonists, or renin inhibitors, or if they were pregnant or currently had a urinary tract infection.

After obtaining informed consent, a spot urine sample was collected from each subject, and a blood sample was drawn for measurement of serum creatinine (Cr) and determination of estimated glomerular filtration rate (eGFR), using the Modification of Diet in Renal Disease (MDRD) calculation [15]. Demographic information was obtained via interview with the subject and/or from the hospital chart. For transplant recipients, a diagnosis of diabetes mellitus and the primary renal diagnoses were obtained directly from survey of the hospital chart.

### Measurement of Urinary Albumin/Creatinine

Urine samples were placed on ice, aliquoted and then centrifuged at 12000 g for 5 min at 4°C. Measurements of urinary albumin were performed on supernatant fractions, using an ELISA kit (Exocell Inc., Philadelphia, PA, USA). Results were corrected for urinary Cr concentration, using a kit specific for human Cr (Creatinine Companion, Exocell Inc.).

### Urinary ACE2 and ACE Enzyme Activity Assays

The enzyme activities of urinary ACE2 and ACE were measured using synthetic substrates, essentially as we previously reported [16]. All results were corrected for the Cr concentration in the urine samples. Details on the assays can be found in Text S1.

### Urinary ACE2 ELISA

The amount of ACE2 present in urine specimens was quantified using a commercial ELISA kit (Cat. No. AG-45A-0022EK-KI01, AdipoGen, Seoul, Korea) according to the protocol provided by the supplier (http://www.adipogen.com/ag-45a-0022/ace2-human-elisa-kit.html). A standard curve was generated by performing 1∶2 serial dilutions of human recombinant ACE2 (50 ng/ml), provided with the kit, with the limit of detection ranging from 0.391 to 25 ng/mL. In preliminary experiments, the average intra-assay coefficient of variation (CV) for the assay was 2.9%, and the average inter-assay CV value was 8.7% (n = 10). The amount of ACE2 obtained by ELISA was normalized to the subject’s urine Cr concentration, and is reported as ng/mg Cr.

### Urinary ACE2 and ACE Immunoblot Assays

Urine aliquots (supernatant fraction, 15 µL) were subjected to immunoblot analysis for ACE2 and ACE, using commercially available antibodies. To control for variations in urine concentration, the values obtained by densitometry were divided by the corresponding Cr concentration for that urine sample. Details on the assays can be found in Text S1.

### Peptide *N*-Glycosidase F (PNGase F) Treatment

Urine aliquots (supernatant fraction, 20 µL) were subject to deglycosylation reaction using PNGase F (Cat No. P0704S, New England Biolabs, Ipswich, MA, USA). Deglycosylated urinary ACE2 protein fragments were detected by western analysis. Details of the assays can be found in Text S1.

### Urinary mRNA Assays

Urine samples (40 mL) were centrifuged at 1000 g for 20 min at 4°C. Total RNA was isolated from pellet fractions and then subjected to real-time RT-PCR for quantitation of ACE2 and ACE (see Text S1 for assay details).

### Urinary Ang II and Ang-(1-7) Assays

Urinary levels of Ang II were measured using a commercial peptide radioimmunoassay (RIA) kit that contains an Ang II-selective polyclonal antibody and ^125^I-Ang II (Peninsula Laboratories, San Carlos, CA, USA), essentially as described [16], [17]. Ang-(1-7) levels were measured using a commercial peptide enzyme immunoassay (EIA) kit that contains an Ang-(1-7)-selective polyclonal antibody (Peninsula Laboratories) [16]. For assay details, see Text S1.

### Analysis

Data are presented visually as box plots. Continuous variables are reported as the median value, with the interquartile range in parentheses. Statistical analysis was performed by the nonparametric Mann-Whitney *U* test for unpaired samples to compare data in healthy controls and transplant recipients, and diabetic vs non-diabetic transplant recipients. A linear regression model was constructed to identify potential explanatory variables for urinary ACE2 and ACE levels in the 100 transplant subjects. The dependent variable was the measure under study (e.g. ACE2 activity) while the following explanatory variables were entered into the model: age, gender, diabetes, eGFR, albuminuria, hypertension, and use of calcineurin inhibitors. Variables were forced in simultaneously and removed from the model if not statistically significant. Standardized regression coefficients (β) are presented for each dependent variable in the model. Data were analyzed using SigmaStat (version 3.01 A; SYSTAT) and JMP (version 8.0.1, SAS Inc.). For all data, a p value <0.05 was considered significant.

## Results

### Patient Demographics

The characteristics of the 50 healthy controls and 100 transplant subjects are depicted in Table 1. The median age for control subjects was 45 [interquartile range (IQR), 36–50] yrs, and 26% were male. The median age for transplant subjects was 54 (IQR, 42–62) yrs, 65% were male, 38% had diabetes, and 88% had hypertension. Median time from transplant was 3 (IQR, 1–7) yrs. The median eGFR was 79.0 (IQR, 69.0–86.0) mls/min/1.73 m^2^ in control subjects and 48.5 (IQR, 40.0–61.0) mls/min/1.73 m^2^ in transplant subjects. Median albuminuria level was 0.94 (IQR, 0.67–1.43) mg/mmol Cr in control subjects and 1.58 (IQR, 0.61–20.63) mg/mmol Cr in transplant subjects. Most transplant patients (89%) were on triple immunosuppressant therapy comprised of prednisone, a calcineurin inhibitor (tacrolimus or cyclosporine), and either azathioprine or mycophenolate mofetil.

**Table 1:** Subject demographic data.

	Control (n = 50)	Patients (n = 100)
Age (years)	45 (36–50)	54 (42–62)
Male gender (%)	26	65
eGFR (mls/min/1.73 m^2^)	79.0 (69.0–86.0)	48.5 (40.0–61.0)
ACR (mg/mmol)	0.94 (0.67–1.43)	1.58 (0.61–20.63)
Diabetes %	0	38
Hypertension %	0	88
Years post-transplant	N/A	3 (1–7)
**Primary cause of renal** **disease, %**		
Diabetic nephropathy	N/A	21
Hypertension	N/A	9
Polycystic kidney disease	N/A	13
Glomerulonephritis	N/A	41
Other	N/A	16
**Triple immunosuppression** **use^a^ %**	N/A	89
CNI (Cyclosporine or Tacrolimus) %	N/A	95
Prednisone %	N/A	97
Mycophenolate mofetil %	N/A	90
Azathioprine %	N/A	8
Sirolimus %	N/A	3

Values are medians with interquartile range in parentheses. Abbreviations: eGFR: estimated glomerular filtration rate, ACR: urine albumin to Cr ratio, CNI: calcineurin inhibitor. ^a^ calcineurin inhibitor, mycophenolate mofetil or azathioprine, and corticosteroid. N/A: not applicable.

### Urinary ACE2 Activity, Protein, and mRNA

**Figure 1:**
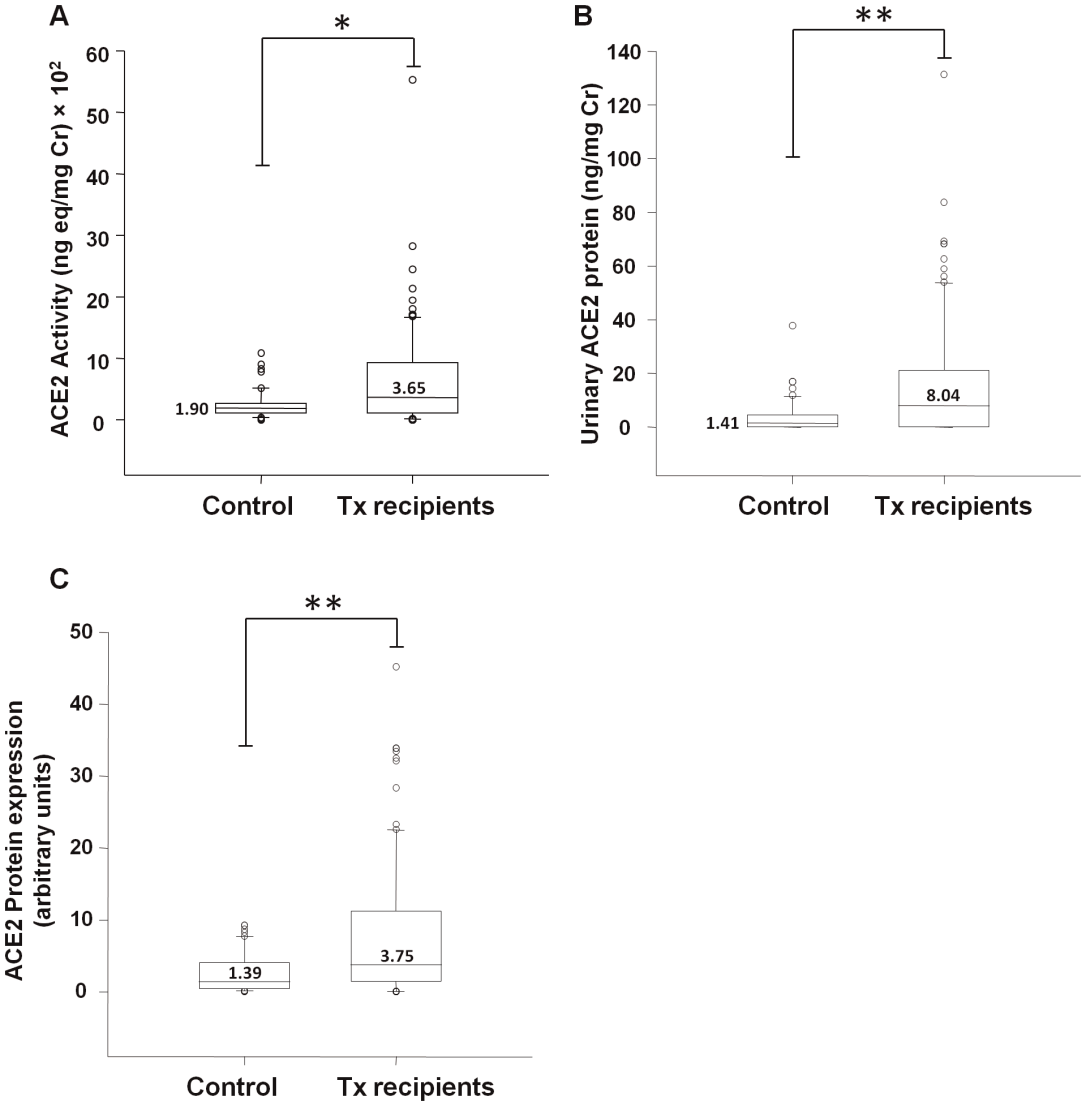
Urinary ACE2 activity and protein in control subjects and renal transplant (Tx) recipients. (A) Graph depicts box plots of urinary ACE2 activity in control subjects (n = 50) and Tx recipients (n = 100). For each box plot, median values are indicated by the line within the box, with value shown beside or above the line. The box represents 50% of the values (25^th^ and 75^th^ percentiles), with the upper bar representing the 90^th^ percentile and the lower bar representing the 10^th^ percentile. Open circles indicate outliers. * p = 0.003, Control vs Tx recipients. (B) Graph depicts box plots of urinary ACE2 protein by ELISA in control subjects and Tx recipients. ** p<0.001, Control vs Tx recipients. (C) Graph depicts box plots of urinary ACE2 protein by western analyses in control subjects and Tx recipients. Densitometry analysis was performed on both urinary ACE2 bands (120 kDa and 90 kDa), and the sum of the two bands was used for quantitative comparisons. **p = 0.001, Control vs Tx recipients.

As shown in Figure 1A, urinary ACE2 activity was significantly increased in transplant patients, compared to control subjects [control; median: 1.90 (IQR, 1.04–2.70) ng-eq/mg Cr×10^2^ vs transplants; median: 3.65 (IQR, 1.13–9.35) ng-eq/mg Cr×10^2^; p = 0.003]. Similarly urinary ACE2 protein by ELISA was significantly increased in transplant patients, compared to controls (Figure 1B) [control; median: 1.41 (IQR, 0.00–4.49) ng/mg Cr vs transplants; median: 8.04 (IQR, 0.00–21.11) ng/mg Cr; p<0.001]. In 96% of unconcentrated urine samples from control and 94% from transplant subjects, ACE2 was identified by western analysis as a protein doublet of 120 kDa and 90 kDa. As with urinary ACE2 activity and ELISA measurements, western analyses revealed a significant increase in urinary ACE2 protein in transplant patients compared to controls (Figure 1C: p = 0.001).

Within the group of 100 transplant patients, multiple linear regression using primary renal diseases as explanatory variables revealed a significant association between the diagnosis of diabetic nephropathy (n = 21) and urinary ACE2 protein by ELISA or western analyses (p<0.001 for both). In contrast, there was no significant association between urinary ACE2 levels and other primary causes of renal disease in these patients. Further studies were performed on the association between diabetes mellitus and urinary ACE2 in the transplant patients. Of the 100 transplant patients, 38 had a diagnosis of diabetes mellitus listed on their hospital chart. The majority of these patients were taking insulin (31 out of 38, 81.6%), 6 (15.8%) were taking oral hypoglycemic agents, and only 1 patient (2.6%) was on dietary therapy alone.

**Figure 2:**
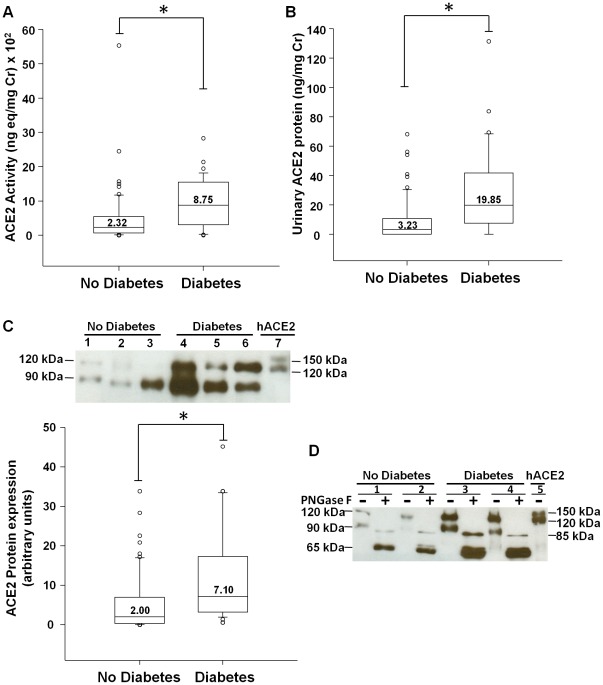
Urinary ACE2 activity and protein in renal transplant recipients: Effect of diabetes. (A) Graph depicts box plots of urinary ACE2 activity in transplant recipients without diabetes (No Diabetes), or with diabetes (Diabetes). For each box plot, median values are indicated by the line within the box, with value shown above the line. The box represents 50% of the values (25^th^ and 75^th^ percentiles), with the upper bar representing the 90^th^ percentile and the lower bar representing the 10^th^ percentile. Open circles indicate outliers. * p<0.001, Diabetes vs. No Diabetes; n = 62 (No Diabetes), n = 38 (Diabetes). (B) Graph depicts box plots of urinary ACE2 protein by ELISA in transplant recipients without diabetes (No Diabetes), or with diabetes (Diabetes). *p<0.001, Diabetes vs. No Diabetes. (C) Graph depicts box plots of urinary ACE2 protein by western analysis in transplant patients without diabetes (No Diabetes), or with diabetes (Diabetes). *p<0.001, Diabetes vs. No Diabetes. Above graph is representative immunoblot for ACE2 in urine, showing bands at 120 kDa and 90 kDa. Densitometry analysis was performed on both bands, and the sum of the two bands was used for quantitative comparisons. The protein bands for ACE2 in urine specimens were not observed when membranes were incubated with the secondary antibody alone, bypassing the primary antibody step. Lanes 1–3, No Diabetes. Lanes 4–6, Diabetes. Lane 7: recombinant human ACE2 protein (hACE2), used as a positive control. (D) Representative immunoblot for urinary ACE2 treated without (−) or with (+) the deglycosylase enzyme PNGase F. Lanes 1+, 2+, 3+, and 4+ show a reduction in the sizes of urinary ACE2 fragments to ∼85 kDa and ∼65 kDa in urine samples treated with PNGase F. Lanes 1 and 2, No Diabetes. Lanes 3 and 4, Diabetes. Lane 5: recombinant human ACE2 protein (hACE2).

Transplant patients with diabetes (n = 38) had significantly higher levels of urinary ACE2 activity, compared to non-diabetic (n = 62) subjects [Figure 2A: diabetics; 8.75 (IQR, 3.09–15.54) vs non-diabetics; 2.32 (IQR, 0.63–5.37) ng-eq/mg Cr×10^2^; p<0.001]. Similarly, ACE2 protein levels by ELISA and western analysis were significantly increased in diabetic subjects, compared to non-diabetics (Figure 2B, C; p<0.001 vs non-diabetics for both). In transplant patients with diabetes, both the 120 kDa and the 90 kDa ACE2 protein bands were significantly increased on westerns, compared to non-diabetics (p = 0.002 vs non-diabetics for the 120 kDa band, and p<0.001 vs non-diabetics for the 90 kDa band). Moreover, urinary ACE2 protein levels by ELISA were significantly increased in subjects with diabetes pre-transplant (n = 21), compared to subjects who developed diabetes after transplant (n = 17; p = 0.024).

The state of glycosylation of the urinary ACE2 proteins at 120 kDa and 90 kDa was studied by treating urine samples with the deglycosylase enzyme PNGase F. Urinary ACE2 fragment sizes were reduced to ∼85 kDa and ∼65 kDa by PNGase F treatment, indicating that both fragments were originally glycosylated (Figure 2D).

RNA was extracted from urinary pellets and subjected to real-time PCR for ACE2. ACE2 mRNA was detected in 45% of urine samples of transplant patients, with no significant difference between diabetic [0.669 (IQR, 0.00–6.15) pg mRNA/mg Cr×10^−5^] and non-diabetic subjects [0.00 (IQR, 0.00–2.06) pg mRNA/mg Cr×10^−5^; p = 0.091].

After adjusting for potential confounding factors [age, gender, diabetes, eGFR, albuminuria, hypertension, and use of calcineurin inhibitors] only diabetes was significantly associated with urinary ACE2 activity (p = 0.003) and protein levels (p<0.001, Table 2) in transplant patients. An increase in urinary ACR was associated with urinary ACE2 protein by ELISA (p = 0.026), but not with ACE2 activity or ACE2 protein by western. Female gender was associated with higher urinary ACE2 mRNA levels in transplant patients (p = 0.004, Table 2).

**Table 2:** Multiple linear regression adjusting for common variables in transplant recipients.

Dependent Variable	Independent Variable	Standardized Coefficient (β)	95% CI	p value
ACE2 Activity				
	Diabetes	0.295	0.104, 0.487	0.003
ACE2 ELISA				
	Diabetes	0.365	0.183, 0.547	<0.001
	ACR	0.207	0.025, 0.389	0.026
ACE2 Western				
	Diabetes	0.332	0.143, 0.521	<0.001
ACE2 mRNA				
	Gender (Female)	0.287	0.095, 0.479	0.004
ACE activity	(none)			
ACE Western				
	ACR	0.327	0.138, 0.516	<0.001
ACE mRNA				
	Gender (Female)	0.222	0.033, 0.411	0.022
	ACR	0.267	0.078, 0.456	0.006
Ang II				
	Diabetes	0.204	0.008, 0.400	0.042
Ang-(1-7)	(none)			

All analyses adjusted for eGFR, age, gender, albuminuria, diabetes, hypertension, and use of calcineurin inhibitors. Abbreviations: eGFR: estimated glomerular filtration rate, ACR: urine albumin to Cr ratio.

Since the numbers of patients taking calcineurin inhibitors (95/100 patients) were highly skewed (Table 1), multivariate analysis was also performed with exclusion of this variable. This did not alter the significance levels for any parameters reported in Table 2.

### Urinary ACE Activity, Protein, and mRNA

**Figure 3:**
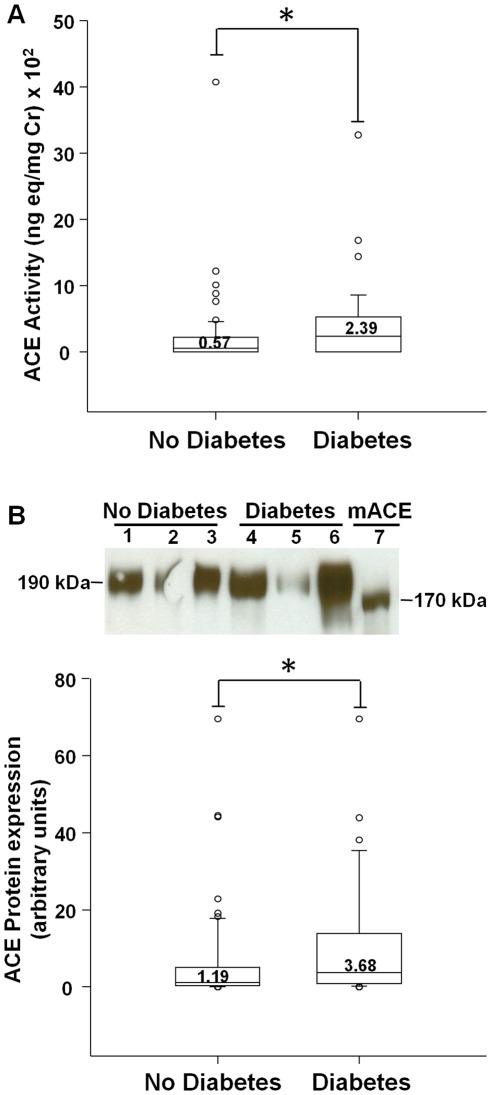
Urinary ACE activity and protein in renal transplant recipients: Effect of diabetes. (A) Graph depicts box plots of urinary ACE activity in transplant recipients without diabetes (No Diabetes) or with diabetes (Diabetes). For each box plot, median values are indicated by the line within the box, with value shown above the line. The box represents 50% of the values (25^th^ and 75^th^ percentiles), with the upper bar representing the 90^th^ percentile and the lower bar representing the 10^th^ percentile. Open circles indicate outliers. * p = 0.013, Diabetes vs No Diabetes. (B) Graph shows box plots for densitometric analysis of protein bands for urinary ACE, at 190 kDa, in transplant recipients without diabetes (No Diabetes) or with diabetes (Diabetes). * p = 0.019, Diabetes vs No Diabetes. Representative ACE immunoblot on urine specimens is shown above graph. In samples from mouse cortex, ACE appeared as a single band at 170 kDa, as expected (mACE, lane 7) (5). This blot was not used for densitometric quantitation of urinary ACE. Protein bands corresponding to ACE were absent when membranes were incubated with the secondary antibody alone. Lanes 1–3, No Diabetes. Lanes 4–6, Diabetes. Lane 7: mouse kidney cortex, used as a positive control.

Urinary ACE activity was detected in 65% of transplant patients [median for all 100 patients: 1.17 (IQR, 0.00–3.43) ng-eq/mg Cr×10^2^]. By western analysis, ACE was detected in 94% of urine samples of transplant patients as a protein band of approximately 190 kDa, consistent with previous studies [18] (Figure 3). Urinary ACE activity and protein levels were significantly increased in diabetic transplant subjects, compared to non-diabetic patients (Figure 3A,B; p = 0.013, p = 0.019, respectively). ACE mRNA was detected in 65% of urine samples of transplant patients, with no significant difference between diabetic [3.06 (IQR, 0.04–19.09) pg mRNA/mg Cr×10^−5^] and non-diabetic patients [0.77 (IQR, 0.00–13.16) pg mRNA/mg Cr×10^−5^; p = 0.18].

After adjusting for potential confounding variables, increased urinary albumin/Cr was associated with urinary ACE protein by western analysis in transplant patients (p<0.001, Table 2). However, diabetes did not associate with increased urinary ACE activity or ACE protein. Female gender and urinary albumin/Cr were significantly associated with increased urinary ACE mRNA levels in transplant patients (Table 2).

### Urinary Ang II and Ang-(1-7)

**Figure 4:**
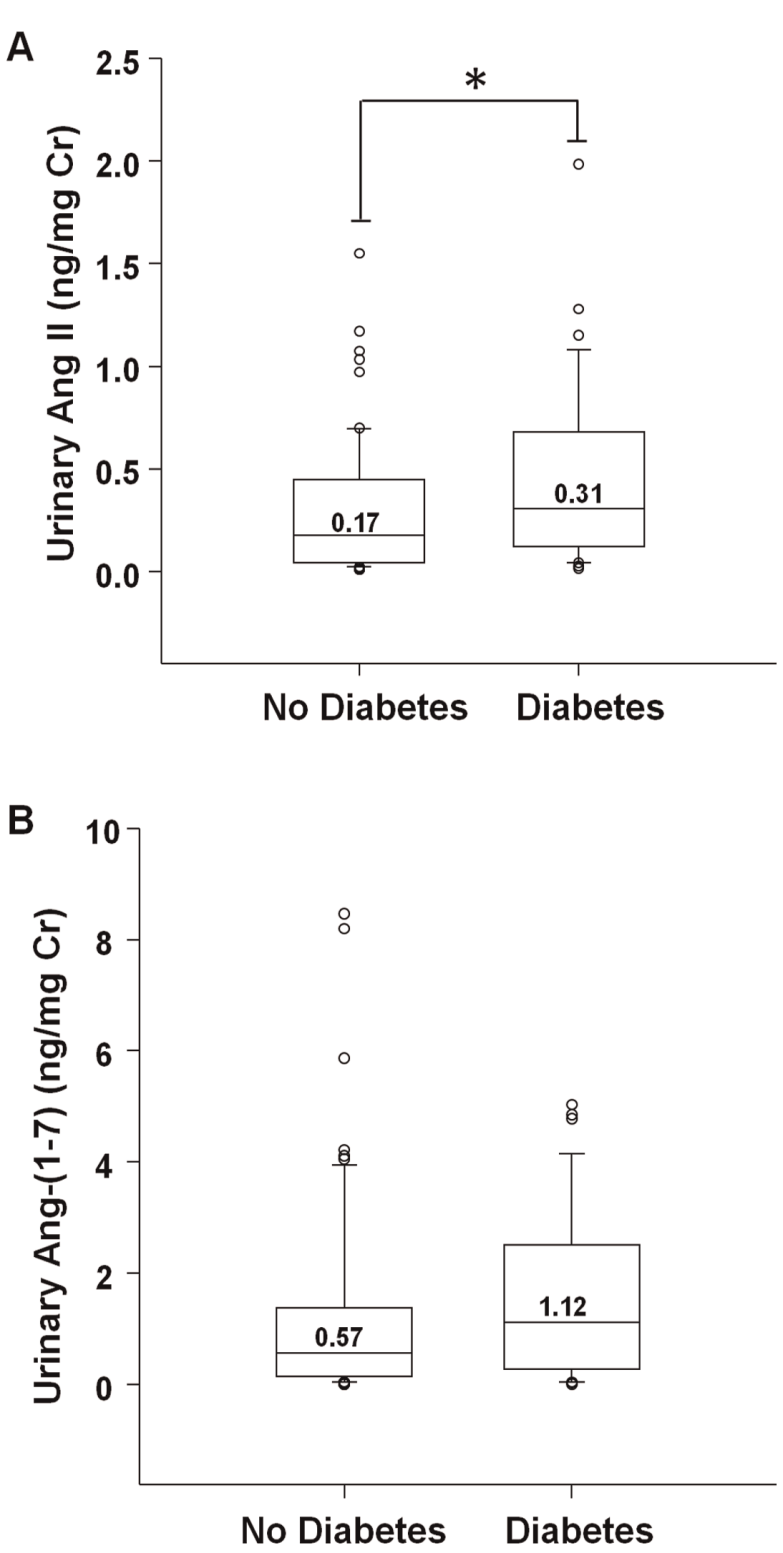
Urinary Ang II and Ang-(1-7). (A) Graph shows box plots of RIA for Ang II in urine specimens from transplant recipients without diabetes (no Diabetes) or with diabetes (Diabetes). For each box plot, median values are indicated by the line within the box, with value shown above the line. The box represents 50% of the values (25^th^ and 75^th^ percentiles), with the upper bar representing the 90^th^ percentile and the lower bar representing the 10^th^ percentile. Open circles indicate outliers. * p = 0.027, Diabetes vs. No Diabetes. (B) Graph shows box plots of EIA for Ang-(1-7) in urine specimens from transplant recipients without or with diabetes. There was no significant difference between the two groups (p = 0.126).

Transplant patient with diabetes had significantly higher levels of urinary Ang II, compared to non-diabetics (Figure 4A: p = 0.027). Urinary Ang-(1-7) levels did not differ between the two groups (Figure 4B: p = 0.13). However, a significant positive linear correlation was found between urinary ACE2 activity and urinary Ang-(1-7) (r = 0.260, p = 0.009). In the multivariate analysis, diabetes remained significantly associated with urinary Ang II levels (p = 0.042, Table 2). Neither diabetes, nor any other clinical factor (eGFR, urinary albumin/Cr, etc.) was associated with urinary Ang-(1-7) levels in the multiple linear regression model.

## Discussion

The major finding of this study is that urinary ACE2 activity and ACE2 protein are increased in kidney transplant recipients, compared to healthy control subjects, and the presence of diabetes strongly associates with urinary ACE2 levels in the patient population, by multivariate analysis. Female gender associates significantly with urinary ACE2 mRNA and ACE mRNA levels, which are detected in 45% and 65% of transplant recipients, respectively.

ACE2 is expressed at relatively high levels in the kidney, particularly in the proximal tubule, and is thought to be renoprotective via its ability to reduce Ang II levels. ACE2 also increases production of Ang-(1-7), which can oppose the deleterious non-hemodynamic actions of Ang II in tubular cells [19]. Kidney expression of ACE2 is reduced in experimental models, including the remnant kidney model of CKD [16], [20], [21]. In humans with diabetic nephropathy, expression of ACE2 is decreased in glomeruli and proximal tubules [22], suggesting a predisposition to Ang II-mediated injury.

Our results support the hypothesis that transplant patients with diabetes exhibit increased urinary excretion of ACE2 protein. This was independent of eGFR as determined by multivariate analysis. Both soluble ACE2 and ACE have been detected in human urine [10], and in humans with CKD, Mizuiri et al. reported increased urinary levels of ACE2 protein by ELISA, compared to healthy subjects [14]. Patients with diabetic nephropathy had higher urinary levels of ACE2 than those without diabetic nephropathy, and use of ACE inhibitor and/or angiotensin receptor blockers did not affect urinary ACE2 levels [14]. In our study, no subjects were taking inhibitors of the RAS. Using three independent assays for urinary ACE2 (enzyme activity, ELISA, and immunoblot), multiple linear regression revealed that diabetes was the only variable consistently predictive of higher urinary ACE2 levels. Age and gender had no influence on urinary ACE2 activity or protein levels. Similarly, in patients with CKD Mizuiri et al. found no difference in urinary ACE2 levels between males and females [14].

Although gender did not influence urinary ACE2 activity or protein, in the present studies females had significantly higher urinary ACE2 mRNA levels. The gene for ACE2 is located on the X chromosome [2], and in the rat renal wrap model of hypertension, intrarenal ACE2 activity and expression are up-regulated by estrogens [23], raising the possibility that female transplant recipients may express ACE2 mRNA at higher levels in the kidney compared to males. Female gender was also associated with higher urinary ACE mRNA levels, as was albuminuria, the latter being in agreement with studies in patients with diabetic nephropathy [24]. The cellular origin of urinary mRNA for ACE2 or ACE is unknown, although it has been speculated that proximal tubular cells may be the source, since they express all components of the RAS, and are found in the urine sediment [24]. However, the physiological significance of urinary ACE2 or ACE mRNA levels is unclear. In this regard, diabetes, albuminuria and eGFR did not influence urinary ACE2 mRNA levels in our transplant recipients, a result that differs from diabetic nephropathy patients, where proteinuria was found to correlate positively with urinary ACE2 mRNA, and eGFR to correlate negatively with ACE2 mRNA [24]. Factors related to transplant could therefore have an independent influence on urinary ACE2 mRNA. As one possibility, the use of immunosuppressive drugs in transplant subjects might regulate shedding of cells expressing ACE2 mRNA into the urine.

Urinary ACE2 protein could originate at least partly from plasma (via filtration at the glomerulus), or it could be derived via excretion from renal cells. Although Mizuiri et al. found higher urinary ACE2 levels in CKD patients, there was no difference in plasma ACE2 protein between CKD patients and healthy subjects [14]. However, the CKD patients in their study had significant albuminuria, suggesting that ACE2 may have leaked into the urine across the glomerular barrier. In the present study, we found no association between albuminuria and either urinary ACE2 activity or protein levels by western analysis. The data suggest that urinary ACE2 protein likely derives via shedding from cells along the nephron, and not from filtration from plasma. In support of this hypothesis, soluble ACE2 is shed from the plasma membrane in cell culture systems, via cleavage at its ectodomain by the protease enzyme ADAM-17 [11]–[13]. Interestingly, renal ADAM-17 is up-regulated in Ang II-induced kidney injury [25], and *de novo* expression occurs in human kidney disease, in proximal tubule, podocytes, and mesangial cells [26]. Whether ADAM-17 is activated in the transplant kidney in the presence of diabetes, and mediates shedding of soluble ACE2 into the urine requires further study.

Two bands for ACE2 were detected by immunoblot of urine samples in the current study. One band was found at 120 kDA, consistent with the full-length protein, and the other at 90 kDa, which is likely a cleaved ACE2 fragment. Thus, the 90 kDa fragment does not simply represent a deglycosylated form of full-length ACE2, since incubation with PNGase F resulted in a further reduction in its size, as shown in Figure 2D. To determine if this 90 kDa fragment is generated by ADAM-17-mediated cleavage of ACE2, further characterization is required, including use of antibodies directed against the cytoplasmic carboxyterminus of ACE2 (which may be lacking in this fragment), and/or direct sequence analysis of the polypeptide. Furthermore, although shedding of ACE2 fragments may occur via ADAM-17 in transplant subjects, proteomic analysis has identified ACE2 as one of the 1132 proteins present in urinary exosomes isolated from human urine [27], suggesting that membrane-bound full-length ACE2 may also be a source of urinary ACE2.

By multivariate analysis, diabetes was significantly associated with urinary Ang II levels in transplant subjects, consistent with intrarenal RAS activation in diabetes [28]. In this regard, although diabetic patients had significantly higher urinary ACE activity and ACE protein levels by immunoblot, this association was not confirmed in the multivariate analysis. Finally, although diabetics had higher urinary ACE2 activity and protein levels, there was no significant association between diabetes and urinary levels of Ang-(1-7) by multivariate analysis, suggesting that urinary Ang-(1-7) may be influenced by other factors. A potential limitation of the urinary angiotensin peptide measurements is that urine specimens were not treated with protease inhibitors, which could have affected the sensitivity for detecting differences. Interestingly, however, we did observe a significant correlation between urinary ACE2 activity and Ang-(1-7) levels.

Our study has a number of strengths, including the reliability and reproducibility of the assays, the inclusion of data on mRNA, protein, and ACE2/ACE products, and the use of multiple linear regression to adjust for confounding variables. Limitations include the relatively small number of subjects, the absence of plasma ACE2 or ACE measurements, and the single time point for urinary ACE2, ACE and peptide assays. Furthermore, it is possible that diabetes alone may contribute to increased urinary ACE2 levels, independent of transplant status. Studies in patients with diabetes and normal kidney function are required to answer this question. Larger studies are also needed to determine if urinary ACE2 or ACE are biomarkers of transplant function or if they may predict responsiveness to blockade of the RAS.

In summary, in renal transplant recipients diabetes is a strong independent predictor of increased urinary levels of ACE2 activity and protein. Our data further suggest that ACE2 may be shed into the urine in transplant recipients, and could represent a marker to assess the role of the kidney RAS in these patients.

## Supporting Information

Text S1 **Detailed methods for enzyme activity assays, immunoblots, real-time PCR assays, and measurements of Ang II and Ang-(1-7).**
(DOC)

## References

[1] Donoghue M, Hsieh F, Baronas E, Godbout K, Gosselin M (2000). A novel angiotensin-converting enzyme-related carboxypeptidase (ACE2) converts angiotensin I to angiotensin 1–9.. Circ Res.

[2] Tipnis SR, Hooper NM, Hyde R, Karran E, Christie G (2000). A human homolog of angiotensin-converting enzyme. Cloning and functional expression as a captopril-insensitive carboxypeptidase.. J Biol Chem.

[3] Li N, Zimpelmann J, Cheng K, Wilkins JA, Burns KD (2005). The role of angiotensin converting enzyme 2 in the generation of angiotensin 1–7 by rat proximal tubules.. Am J Physiol Renal Physiol.

[4] Shaltout HA, Westwood BM, Averill DB, Ferrario CM, Figueroa JP (2007). Angiotensin metabolism in renal proximal tubules, urine, and serum of sheep: evidence for ACE2-dependent processing of angiotensin II.. Am J Physiol Renal Physiol.

[5] Ye M, Wysocki J, Naaz P, Salabat MR, LaPointe MS (2004). Increased ACE 2 and decreased ACE protein in renal tubules from diabetic mice: a renoprotective combination?. Hypertension.

[6] Oudit GY, Herzenberg AM, Kassiri Z, Wong D, Reich H (2006). Loss of angiotensin-converting enzyme-2 leads to the late development of angiotensin II-dependent glomerulosclerosis.. Am J Pathol.

[7] Wong DW, Oudit GY, Reich H, Kassiri Z, Zhou J (2007). Loss of angiotensin-converting enzyme-2 (Ace2) accelerates diabetic kidney injury.. Am J Pathol.

[8] Wysocki J, Ye M, Rodriguez E, González-Pacheco FR, Barrios C (2010). Targeting the degradation of angiotensin II with recombinant angiotensin-converting enzyme 2: prevention of angiotensin II-dependent hypertension.. Hypertension.

[9] Oudit GY, Liu GC, Zhong J, Basu R, Chow FL (2010). Human recombinant ACE2 reduces the progression of diabetic nephropathy.. Diabetes.

[10] Warner FJ, Lew RA, Smith AI, Lambert DW, Hooper NM (2005). Angiotensin-converting enzyme 2 (ACE2), but not ACE, is preferentially localized to the apical surface of polarized kidney cells.. J Biol Chem.

[11] Iwata M, Silva Enciso JE, Greenberg BH (2009). Selective and specific regulation of ectodomain shedding of angiotensin-converting enzyme 2 by tumor necrosis factor alpha-converting enzyme.. Am J Physiol Cell Physiol.

[12] Lambert DW, Yarski M, Warner FJ, Thornhill P, Parkin ET (2005). Tumor necrosis factor-alpha convertase (ADAM17) mediates regulated ectodomain shedding of the severe-acute respiratory syndrome-coronavirus (SARS-CoV) receptor, angiotensin-converting enzyme-2 (ACE2).. J Biol Chem.

[13] Jia HP, Look DC, Tan P, Shi L, Hickey M (2009). Ectodomain shedding of angiotensin converting enzyme 2 in human airway epithelia.. Am J Physiol Lung Cell Mol Physiol.

[14] Mizuiri S, Aoki T, Hemmi H, Arita M, Sakai K (2011). Urinary ACE2 in patients with CKD.. Nephrology (Carlton).

[15] Levey AS, Coresh J, Greene T, Stevens LA, Zhang YL (2006). Using standardized serum creatinine values in the modification of diet in renal disease study equation for estimating glomerular filtration rate.. Annal Int Med.

[16] Dilauro M, Robertson S, Genest D, Burns KD (2010). The effect of ACE2 and angiotensin-(1–7) in a mouse model of early chronic kidney disease.. Am J Physiol Renal Physiology.

[17] Hermann K, Rittweger R, Phillips MI, Ring J (1992). Presence of angiotensin peptides in human urine.. Clin Chem.

[18] Casarini DE, Plavinik FL, Zanella MT, Marson O, Krieger JE (2001). Angiotensin converting enzymes from human urine of mild hypertensive untreated patients resemble the N-terminal fragment of human angiotensin I-converting enzyme.. Int J Biochem Cell Biol.

[19] Su Z, Zimpelmann J, Burns KD (2006). Angiotensin-(1–7) inhibits angiotensin II-stimulated phosphorylation of MAP kinases in proximal tubular cells.. Kidney Int.

[20] Koitka A, Cooper ME, Thomas MC, Tikellis C (2008). Angiotensin converting enzyme 2 in the kidney.. Clin Exp Pharmacol Physiol.

[21] Leehey DJ, Singh AK, Bast JP, Sethupathi P, Singh R (2008). Glomerular renin angiotensin system in streptozotocin diabetic and Zucker diabetic fatty rats.. Transl Res.

[22] Reich HN, Oudit GY, Penninger JM, Scholey JW, Herzenberg AM (2008). Decreased glomerular and tubular expression of ACE2 in patients with type 2 diabetes and kidney disease.. Kidney Int.

[23] Ji H, Menini S, Zheng W, Pesce C, Wu X (2008). Role of angiotensin-converting enzyme 2 and angiotensin(1–7) in 17beta-oestradiol regulation of renal pathology in renal wrap hypertension in rats.. Exp Physiol.

[24] Wang G, Lai FM, Lai KB, Chow KM, Kwan CH (2008). Urinary mRNA expression of ACE and ACE2 in human type 2 diabetic nephropathy.. Diabetologia.

[25] Lautrette A, Li S, Alili R, Sunnarborg SW, Burtin M (2005). Angiotensin II and EGF receptor cross-talk in chronic kidney diseases: a new therapeutic approach.. Nature Med.

[26] Melenhorst WB, Visser L, Timmer A, van den Heuvel MC, Stegeman CA (2009). ADAM17 upregulation in human renal disease: a role in modulating TGF-alpha availability?. Am J Physiol Renal Physiol.

[27] Gonzales PA, Pisitkun T, Hoffert JD, Tchapyjnikov D, Star RA (2009). Large-scale proteomics and phosphoproteomics of urinary exosomes.. J Am Soc Nephrol.

[28] Mezzano S, Droguett A, Burgos ME, Ardiles LG, Flores CA (2003). Renin-angiotensin system activation and interstitial inflammation in human diabetic nephropathy.. Kidney.

