# Supplementary material for: Increased Urinary Angiotensin-Converting Enzyme 2 in Renal Transplant Patients with Diabetes
Source: PLoS One. 2012 May 22;7(5):e37649. doi: 10.1371/journal.pone.0037649 (PMC3358292; doi:10.1371/journal.pone.0037649)
Supplement: Text S1 — Detailed methods for enzyme activity assays, immunoblots, real-time PCR assays, and measurements of Ang II and Ang-(1-7). (DOC) [file pone.0037649.s001.doc]

**Urinary ACE2 and ACE enzyme activity assays**

The enzymatic activity of ACE2 in urine (supernatant fraction) was measured using a commercially available synthetic fluorogenic substrate for ACE2 (Mca-Ala-Pro-Lys(Dnp)-OH) (AnaSpec, San Jose, CA, USA). Briefly, urine aliquots (5 µL) were incubated on 96-well plates in a 100 µL solution containing 50 mM MES, 300 mM NaCl, 10 µM ZnCl_2_, 1 mM *N*-ethylmaleimide, 1 mM phenylmethylsulfonyl fluoride (PMSF), 30 µM of the ACE2 substrate, with or without 1 µM of the ACE2 inhibitor MLN-4760 (GL1001, provided by Ore Pharmaceuticals, Cambridge, MA, USA). Samples were mixed on a plate shaker at room temperature in the dark and fluorescence was measured over 16 h using the FLUOstar Galaxy fluorometer (BMG Labtech., Durham, NC, USA) (excitation 320 nm, emission 405 nm). Blank values were subtracted from all fluorescence values. ACE2 activity was determined by subtracting the absorbance value for the mixture incubated with MLN-4760 from the absorbance measured from the mixture without MLN-4760, as we previously reported [16]. A standard curve was generated for the assay, using purified recombinant mouse ACE2 (R&D Systems Inc., Minneapolis, MN, USA). Results were corrected for the Cr concentration in the urine samples.

A fluorescence-based assay was used for the measurement of urinary ACE activity, which involved incubation of urine aliquots (5 µL) with 0.45 mM of the ACE substrate *o*-aminobenzoylglycyl-*p*-nitro-*L*-phenylalanyl-*L*-proline (Abz-Gly-Phe(NO2)-Pro) (Bachem Biosciences Inc., King of Prussia, PA, USA), in a buffer containing 150 mM Tris (pH 8.3), in the presence or absence of captopril (10 µM) [16]. The compound Abz-Gly-OH-HCl (Bachem Biosciences Inc.) was used to generate standard curves for the assay.

**Urinary ACE2 and ACE immunoblot assays**

Urine aliquots (supernatant fraction, 15 µL) were subjected to immunoblot analysis for ACE2 and ACE, using commercially available antibodies. Details on the assays can be found in the on-line supplement. Samples were prepared in a buffer consisting of 31.3 mM Tris-HCl (pH 6.8), 1% wt/vol SDS, 5% glycerol, and 0.025% wt/vol bromophenol blue. After boiling for 5 min, the samples were centrifuged at 12000 g for 5 min to remove insoluble debris. Samples were then run on 7.5% SDS-polyacrylamide gels and transferred to nitrocellulose membranes (Bio-Rad Laboratories, Mississauga, ON, Canada). The membranes were blocked with 5% skim milk in Tris-buffered saline (pH 7.6) containing 0.1% Tween 20 (TBS-T) for 1 h at room temperature. The membranes were then incubated for 16 h at 4^o^C with a 1:500 dilution of a goat polyclonal antibody to ACE2, reactive to the human protein (AF933, R&D Systems), or with a 1:500 dilution of a goat polyclonal antibody to ACE (AF929, R&D Systems). After incubation with the primary antibody, the membranes were incubated with a 1:2000 dilution of horseradish peroxidase-conjugated secondary antibody (Jackson Immuno-Research Laboratories, West Grove, PA, USA). Proteins were detected by enhanced chemiluminescence (ECL; GE Health Care Bio-Sciences, Baie d’Urfe, Quebec, Canada). Pre-stained standards were used as molecular weight markers (Bio-Rad), and all gels contained samples with either human recombinant ACE2 (R&D Systems) or mouse kidney cortex (for ACE) as controls. Densitometric analysis of the protein bands was performed using Kodak ID image Analysis software (Eastman Kodak, Rochester, NY, USA). To control for variations in urine concentration, the values obtained by densitometry were divided by the corresponding Cr concentration for that urine sample.

**Peptide *N*-glycosidase F (PNGase F) Treatment**

Urine aliquots (supernatant fraction, 20 µL) were deglycosylated using peptide *N*-glycosidase F (PNGase F, Cat No. P0704S, New England Biolabs, Ipswich, MA, USA), according to the manufacturer’s instructions. Urine aliquots were denatured in 0.5% SDS, 40 mM DTT at 100^o^ C for 10 min. After samples were cooled to room temperature, they were incubated in 50 mM sodium phosphate (pH 7.5), 1% NP-40 (v/v), and PNGase F (750 U) at 37^o^ C for 3 h. Deglycosylated urinary proteins were separated by 7.5% SDS-PAGE and ACE2 immunoreactive bands were detected by western analysis as described above.

**Urinary mRNA assays**

Urine samples (40 mL) were centrifuged at 1000 g for 20 min at 4^o^C. Total RNA was isolated from pellet fractions and then subjected to real-time RT-PCR for quantitation of ACE2 and ACE. Briefly, RNA was isolated from pellets using a commercial kit (RNeasy, Qiagen Inc., Toronto, ON, Canada), and then treated with DNAse for 15 min at room temperature, followed by inactivation at 65^o^C for 10 min (Invitrogen Canada Inc., Burlington, ON, Canada) to remove any residual genomic DNA. RNA was then reverse transcribed and subjected to PCR, using a one-step master mix (TaqMan Gene Expression Assays, Applied Biosystems Inc., Foster City, CA, USA) that contained primers for either human ACE2 or human ACE (Applied Biosystems Inc., catalogue no. Hs01085333 and Hs01104599, respectively). Real-time PCR was performed with an ABI 7000 Sequence Detection System (Applied Biosystems, Inc.), with initial denaturing of samples for 10 min at 95^o^C, followed by 40 cycles of amplification, with denaturing for 15 sec at 95^o^C and annealing/extension for 1 min at 60^o^C. Quantitation of the mRNA of interest was conducted by comparing the cycle numbers needed for the fluorescence of PCR products to reach threshold, and PCR was also performed with primers for human glyceraldehyde-3-phosphate dehydrogenase (GAPDH) (Applied Biosystems Inc., catalogue no. 4310884E-0810040), as an internal control. All experiments included controls in which reverse transcriptase was absent from the reaction mix. Human kidney total RNA was used to generate standard curves for real-time RT-PCR for each mRNA of interest (Clontech, Mountain View, CA, USA). Following mRNA quantitation as the ratio of ACE2 or ACE mRNA to GAPDH mRNA, values were corrected for the Cr concentration in the urine specimen.

**Urinary Ang II and Ang-(1-7) assays**

Urinary levels of Ang II were measured on samples acidified with 7N HCl (8 x10^-3^ vol/vol) using a commercial peptide radioimmunoassay (RIA) kit that contains an Ang II-selective polyclonal antibody and ^125^I-Ang II (Peninsula Laboratories, San Carlos, CA, USA), essentially as described [16,17]. Ang-(1-7) levels were measured using a commercial peptide enzyme immunoassay (EIA) kit that contains an Ang-(1-7)-selective polyclonal antibody (Peninsula Laboratories) [16].

For angiotensin peptides, urine samples were centrifuged at 10000 g for 5 min. Supernatants (500 µL) were mixed with 4 mL of 50 mM sodium phosphate buffer (pH 7.4) containing 0.1% BSA and applied to C18 Sep-Pak columns (Waters Corporation, Milford, MA, USA) that were equilibrated with 2 mL of a buffer consisting of 60% acetonitrile, 1% trifluoroacetic acid (TFA), and 39% distilled water, followed by three washes (3 mL) with 1% TFA. After sample application, each C18 Sep-Pak column was washed twice with 3 mL of 1% TFA. The Ang peptides were eluted in polyproplyene tubes with 2 × 1 mL of the same buffer used to equilibrate the columns. Eluants were concentrated to dryness using a vacuum centrifuge and stored at -80^o^C until the RIA and EIA were performed. Standard curves were generated for each assay, and the results were corrected for the corresponding urinary Cr concentrations.
